# The ISMARA client

**DOI:** 10.12688/f1000research.9794.1

**Published:** 2016-12-15

**Authors:** Panu Artimo, Séverine Duvaud, Mikhail Pachkov, Vassilios Ioannidis, Erik van Nimwegen, Heinz Stockinger

**Affiliations:** 1 SIB Swiss Institute of Bioinformatics, Lausanne, Switzerland; 2 Biozentrum, University of Basel & SIB Swiss Institute of Bioinformatics, Basel, Switzerland

**Keywords:** bioinformatics, data analysis, motif activity response analysis, genome, command line tool, Graphical User Interface

## Abstract

ISMARA ( ismara.unibas.ch) automatically infers the key regulators and regulatory interactions from high-throughput gene expression or chromatin state data. However, given the large sizes of current next generation sequencing (NGS) datasets, data uploading times are a major bottleneck. Additionally, for proprietary data, users may be uncomfortable with uploading entire raw datasets to an external server. Both these problems could be alleviated by providing a means by which users could pre-process their raw data locally, transferring only a small summary file to the ISMARA server. ****We developed a stand-alone client application that pre-processes large input files (RNA-seq or ChIP-seq data) on the user's computer for performing ISMARA analysis in a completely automated manner, including uploading of small processed summary files to the ISMARA server. This reduces file sizes by up to a factor of 1000, and upload times from many hours to mere seconds. The client application is available from ismara.unibas.ch/ISMARA/client.

## Introduction

Motif activity response analysis (MARA) is a general method that models genome-wide expression or chromatin state data in terms of computationally predicted regulatory sites for transcription factors (TFs) and microRNAs to infer the key regulators, their targets, and regulatory interactions between regulators, that are operating in a given system ( Arnold *et al.,* 2013; Balwierz *et al*., 2014; Suzuki *et al*., 2009). MARA has been successfully used to reconstruct core regulatory networks across a wide range of mammalian systems (e.g. see Balwierz *et al*., 2014 and citations therein) and has recently been implemented as a completely automated online system called ISMARA (Integrated System for Motif Activity Response Analysis; ismara.unibas.ch; Balwierz *et al*., 2014). ISMARA is also one of many resources that are part of Switzerland’s Service Delivery Plan in ELIXIR ( www.elixir-europe.org). To run ISMARA, a user only needs to upload her/his raw data to the server, which can be either gene expression data (microarray or RNA-seq data) or chromatin state data (ChIP-seq data) from a set of biological samples. Although ISMARA is a highly popular tool, the current sizes of raw next-generation sequencing datasets are so large (up to hundreds of GBs), that their upload to the web server can require many hours, and this has become a major bottleneck for many users.

To address this problem, we have developed a stand-alone client application (called the ISMARA client) that completely automates the process of pre-processing the user's raw data on her/his own computer, and transmits the much smaller resulting processed files to the ISMARA server for analysis. Since the processed files are many orders of magnitude smaller than the original raw files, the upload is short, even with slow Internet connection speeds.

The resulting processed file (typically several MBs large) is a simple tab-delimited file, which is sent to the ISMARA web server, where it is analyzed in the exactly the same way as when raw data is uploaded. The pre-processing that the ISMARA client performs is also identical to the pre-processing that would otherwise take place on the ISMARA server. Overall, by reducing transfer load and therefore upload times, the ISMARA server is less busy with file transfers, can respond quicker to client requests and the end-user experience is generally improved by shorter waiting times.

Another important feature of the ISMARA client is that it allows users to only communicate highly summarized data to the ISMARA server. In many cases users may be uncomfortable with uploading entire raw datasets of potentially highly competitive data to an external server. By using the ISMARA client, the raw data stays within the premises of users, whereas only small summary information is sent to the ISMARA server for further processing.

## Methods and implementation

In developing the ISMARA client application, our main objectives were to reduce data transfer times and to provide a software application that is easy to install and use on several platforms, i.e., operating systems. We selected the framework Qt5 ( www.qt.io) using QML ( http://doc.qt.io/qt-5/qtqml-index.html) for the user interface and C++ for the platform-independent part. Several of the pre-processing steps that are currently performed on raw data by the ISMARA web server have been implemented on the client side, i.e., within the ISMARA client, and packaged as a native application for Mac OS X and Linux.

The ISMARA client can process microarray data (CEL files), and RNA-seq and ChIP-seq data (BAM/BED files). Depending on the data type there are different processing procedures. For microarray data, the ISMARA client first performs background correction on the probe intensities, followed by correction and adjustment for non-specific binding, and then filters out consistently non-expressed probes. After this, it quantile normalizes the intensities across the samples and log-transforms them. A list of microarray chips that are currently supported is available on the ISMARA website (cf “Usage” at ismara.unibas.ch/fcgi/mara). For RNA-seq data, the client first sorts and indexes the input files, maps the reads to ISMARA's transcript set for the corresponding organism, uses ISMARA's associations between promoters and transcripts and the annotated transcript lengths to calculate normalized expression levels per promoter, and finally log-transforms the expression levels. ChIP-seq data files are sorted and indexed, reads that map to promoter regions (2kb regions centered on each promoter) are counted, the counts are normalized and log-transformed. Detailed descriptions of all processing steps can be found in the original ISMARA paper ( Balwierz *et al*., 2014).

The actual software application uses several external tools, including samtools ( Li *et al*., 2009), htslib and bedops ( Neph *et al*., 2012), as well as scripts and modules in R and Python. Additionally, a new internal interface has been developed on the ISMARA server that is used by the ISMARA client to automatically upload locally pre-processed data.

From a user's point of view, the ISMARA client is a convenient tool that takes large raw data files as input, processes them locally (using several CPU cores in parallel) and then submits the results of the pre-processing as a tab-delimited text file to the ISMARA server. The server then performs MARA on this pre-processed data and displays the final results in a web page, i.e. exactly as when raw data are uploaded to the web server. The user experience of the client and the existing web application are very similar, i.e., the client follows the web site's look and feel. The user starts by selecting the data type (microarray, RNA-seq or ChIP-seq): for RNA-seq and ChIP-seq, the user is also requested to select a genome assembly [human genome versions hg18 or hg19 or mouse genome version 9 (mm9)].

Once the options are selected, a user can add files in CEL, BAM or BED formats. Next, the pre-processing is started by clicking on the “Process data” button. Note that, if present, the “Email” and “Project name” fields can be used by the ISMARA server to send a notification when processing of a specific job has finished.

Additionally, the ISMARA client also implements a new functionality that is currently not available on the web server: several jobs, i.e., processing/submission requests can be managed with the client application. In particular, the client stores all on-going and finished jobs of the user, including their download URLs, so that it is easy to manage multiple sets of experiments. Detailed log information is also available and can be copy-pasted for further communication with the ISMARA team in case of problems or questions.

### Supported platforms and requirements

In order to allow and test for platform-independence, the application was developed on several Linux flavours (Linux Mint, CentOS and Ubuntu), as well as on Mac OS X using bash UNIX shell as the main glue between scripts and external applications. Original plans also included to support MS Windows natively (Qt5 allows that), but external dependencies on scripting and bioinformatics software, such as Python, samtools, R, and Bash, for which support is limited on MS Windows, could not be resolved without considerable re-engineering efforts. Therefore, we decided to use VirtualBox ( www.virtualbox.org) to create disk images that can also be run on Windows machines. In detail, an Ubuntu client image of ISMARA can be run on VirtualBox and installed on MS Windows, allowing Windows users to make use of the ISMARA client.

In summary, easily installable binary applications of the ISMARA client are currently provided on-line for Ubuntu 15.04 and Mac OS X (10.10 and 10.11). Additionally, other Linux flavours and/or virtual machine images via VirtualBox can be provided on demand. The ISMARA client can be installed on a machine with the following modest requirements: 4 GB RAM, and fairly recent versions of R (3.2.0 and 3.1.2 for Mac and Linux, respectively) and Python (2.7.6 and 2.7.9 for Mac and Linux, respectively) need to be preinstalled. Notably, because experimental files can be several tens of GBs large, the client allows machines with limited amounts of disk space to make use of external hard drives. Importantly, usage of an external hard drive has no significant impact on the pre-processing performance and can be easily set up from the ISMARA client’s preferences.

## Results

To assess the performance of the client in comparison with usage of the ISMARA webserver directly we compared two scenarios that we denoted S1 and S2 (cf. Table 1): S1 uses the ISMARA client to pre-process data (P1), uploads small summary files to the server (Upload), and then performs the final analysis on the server (P2). Scenario S2 uploads all data (i.e., large files) to the ISMARA server directly, without using the ISMARA client, and lets the server perform both the pre-processing and final analysis (P1+P2). We tested both scenarios on networks with different speeds and used two different datasets: a set of RNA-seq files (GEO accession, GSE30611) with a total size of 30.2 GB, and a set of ChIP-seq files (GEO accession, GSE26386) with a total size of 3.6 GB.

**Table 1.** Performance results using ISMARA client with three different input datasets. The analysis used a client with 4 cores and a server with 12 cores, on both fast and slow networks. Tests were done in July 2016.

RNA-seq	Network	Upload	P1	Upload	P2	Total
		*30.2 GB*		*17.4 MB*		
S1 client+ server	1 Gbit/s	N/A	95 min	3 s	70 min	**165 min** (2h45)
	10 Mbit/s	N/A	95 min	15 s	70 min	**165 min** (2h45)
S2 server only	1 Gbit/s	30–60 min	35 min	N/A	70 min	**135–165 min**
	10 Mbit/s	360 min	35 min	N/A	70 min	**465 min** (7h45)
ChIP-seq	Network	Upload	P1	Upload	P2	Total
		*3.6 GB*		*10.4 MB*		
S1 client+ server	1 Gbit/s	N/A	8 min	3 s	15 min	**23 min**
	10 Mbit/s	N/A	8 min	13 s	15 min	**23 min**
S2 server only	1 Gbit/s	3–8 min	7–18 min	N/A	15 min	**25–41 min**
	10 Mbit/s	43 min	7–18 min	N/A	15 min	**65–67 min**
Microarray	Network	Upload	P1	Upload	P2	Total
		*39.6 MB*		*64 MB*		
S1 client+ server	1 Gbit/s	N/A	7 min	5 s	22 min	**29 min**
	10 Mbit/s	N/A	7 min	24 s	22 min	**29 min**
S2 server only	1 Gbit/s	5 s	19 min	N/A	40 min	**59 min**
	10 Mbit/s	15 s	19 min	N/A	40 min	**59 min**

### Data transfer size and speed

To investigate the performance gains of the ISMARA client for transferring data of reduced size, we compared the sizes of the original input files with the data file sizes that are obtained from the pre-processing by the client (P1). We analysed expression and ChIP-seq data on middle range desktop machines (Intel core i7 quadcore processors) running Linux Mint or Mac OS X using the example data available on the ISMARA server in the ‘sample data’ section. The pre-processing of ChIP-seq and RNA-seq data on the client lead to file size reductions of a factor of about 300 to more than a 1000 (10.4 MB and 17.4 MB compared to the original file sizes of 3.6 GB and 30.2 GB, respectively). A smaller file size reduces network transfer times significantly ( Stockinger *et al*., 2002), particularly on long low latency wide-area network connections. For the RNA-seq example in Table 1, uploading the original 30.2 GB files took from 30 to 60 min on fast networks (1 Gbit/s network speed) to 5–6 hours on “normal” (mid-size/home network links with 10 Mbit/s speed). In contrast, uploading the pre-processed data file of 17.4 MB took only several seconds on both fast and slow links.

### Total execution speed

Next, we compared end-to-end processing times of scenarios S1 and S2 (cf. column ‘Total’ in Table 1). For the S1 scenario, using 4 cores for the ISMARA client, we observed a total processing time of 2h45 for **RNA-seq**, including client side processing, upload and web server side processing. Upload time was negligible due to the small size of the pre-processed data file. For the S2 scenario, in which 30.2 GB of data is first uploaded to the server before all processing is done on the 12-core ISMARA server, we observed the following two total processing times: 2h15–2h45 for a 1 Gbit/s network and 7h45 for a 10 Mbit/s network. In summary, using the client on 10 Mbit/s (“slower”) networks was always faster than using the server only (S2). Even for fast networks, the observed total processing time was similar for S1 and S2.

For the **ChIP-seq** data ( Table 1), overall execution times of scenarios S1 and S2 were similar. Finally, we did not observe any file size reductions for **microarray** experiments (GEO accession, GSE26386), due to the fact that input file sizes were much smaller (e.g. 36.9 MB) for microarray data in comparison with RNA-seq and ChIP-seq data. Notably, the client pre-processed data files that were uploaded remained relatively small for microarray data as well. Overall, the total processing times for scenarios S1 and S2 with microarray data showed no significant differences.

## Conclusion

The ISMARA client works very well for medium to large datasets by reducing both data transfer times and in many cases also the overall execution times.

## Software availability

ISMARA client available from: https://ismara.unibas.ch/ISMARA/client/

ISMARA client source code: https://gitlab.isb-sib.ch/ST/ismara-client

ISMARA client archived source code at time of publication: DOI, 10.5281/zenodo.192284 ( Artimo *et al*., 2016)

( https://zenodo.org/record/192284#.WEbJSNWLTcs)

Licence: GPL v2
