# Supplementary material for: The ISMARA client
Source: F1000Res. 2016 Dec 15;5:ELIXIR-2851. [Version 1] doi: 10.12688/f1000research.9794.1 (PMC5302150; doi:10.12688/f1000research.9794.1)
Supplement: Peer review history

# Peer review: Referee response for version 1

**DOI:** 10.5256/f1000research.10560.r19973

**Published:** 2017-02-07

**Authors:** Josep Lluís Gelpi

**Affiliations:** 1 Joint BSC - CRG - IRB Programme in Computational Biology, Barcelona Supercomputing Center, University of Barcelona, Barcelona, Spain

The paper reports a client software for the ISMARA server at SIB. The rational of the application is to pre-process data at user’s premises reducing the amount of time required to upload raw data to the server. This is indeed very reasonable and due to the usual size of input data, it represents a significant time saving. The client installs ok and software requirements are reasonable. Also the interface is friendly and easy to follow. Some comments/suggestions  follow:

 1. Instructions to install in a virtual machine for Windows are confusing. Links go to the installation packages of Virtualbox and Ubuntu desktop. I understand that users are expected to install the software in an empty, Ubuntu VM after Virtualbox is available. This needs some skills in system administration. An easier way would be to download a VirtualBox VM with the software already installed. Consider providing such ready-to-run VM, or alternatively a container (Docker or other).

 2.  Data is uploaded automatically after pre-processing.  Does the server calculation also start automatically after upload? Results page should auto-reload when calculation is completed.

 3. Consider making the upload optional (although it can be the default). Users may be interested in checking the intermediate files before running the ismara calculation, and upload manually the relevant ones. Users may also store the intermediate files or re-use them for other analysis.

 4. Although the client is linked to a GUI, presumably, the pre-processing work can be done also from a command line. If this is the case, help on the command line instruction and parameters would be useful. In this way, experienced users could prepare a batch pre-processing job, or perhaps chain this in a larger workflow. Details of the procedure for uploading should be indicated.

 5. Source is made open, but no indication about the policy of contribution is available.

 6. In openSUSE KDE desktop, the interface show some visual problems: Data type menu is cut (Use miRNA does not appear), links on the output are not clickable. Also FAQ and Technical Support links are missing.

 7. URL to the ISMARA results page does not appear in the log file, while text indicates it is.

I have read this submission. I believe that I have an appropriate level of expertise to confirm that it is of an acceptable scientific standard.

# Peer review: Referee response for version 1

**DOI:** 10.5256/f1000research.10560.r18518

**Published:** 2017-01-18

**Authors:** Carsten O. Daub

**Affiliations:** 1 Department of Biosciences and Nutrition, Science For Life Laboratory (SciLifeLab), Karolinska Institutet (KI), Stockholm, Sweden

Artimo et al. present a software tool to pre-process microarray, RNA-Seq and ChIP-Seq data for server-based ISMARA motif activity response analysis. With the novel client tool, the data transfer from the user to the ISMARA server is dramatically reduced saving time and allowing to keep the primary data confidential.

 The developed client tool is a very useful complement to the ISMARA server. It makes the ISMARA server much more user friendly. The manuscript is well written with sufficient level of detail.

 I have two minor suggestions:

1. The client logfile is replaced after each start of the client. It might be helpful to be able to access logfiles for each of the jobs individually as well as even after restarting the client.
2. It was unclear to me to which genome version the sample data was mapped to. It might also help to state the species for the sample data in case a user does not read the GEO entries. 

I have read this submission. I believe that I have an appropriate level of expertise to confirm that it is of an acceptable scientific standard.
